# Idiopathic Thrombocytopenic Purpura after Mastectomy and Axillary Lymph Node Dissection

**DOI:** 10.1155/2014/316064

**Published:** 2014-03-06

**Authors:** Wil L. Santivasi, Meghan M. Routt, Alicia M. Terando

**Affiliations:** Division of Surgical Oncology, Department of Surgery, The Ohio State University College of Medicine, N924 Doan Hall, 410 W 10th Avenue, Columbus, OH 43210, USA

## Abstract

First described in 1916, idiopathic thrombocytopenic purpura (ITP) is an autoimmune disease resulting in the destruction of platelets. Here, we present a case of an 85-year-old patient diagnosed with invasive ductal carcinoma of the breast whose surgical treatment was complicated postoperatively by acute-onset thrombocytopenia with a resultant hematoma at the operative site. Diagnostic Workup revealed no clear etiology for the thrombocytopenia; therefore, a presumptive diagnosis of idiopathic thrombocytopenic purpura was made. Previous literature has associated the development of idiopathic thrombocytopenic purpura with breast cancer. However, to the authors' knowledge, there are no reported cases of ITP presenting immediately following surgical intervention for breast cancer in the absence of other etiologic factors.

## 1. Introduction

Idiopathic thrombocytopenic purpura (ITP) is an autoimmune disease, first described in 1916 [1], in which autoantibody production results in destruction of platelets and thrombocytopenia [2]. Prior reports of ITP in patients diagnosed with breast cancer exist where ITP preceded the diagnosis of breast cancer [3] or occurred during adjuvant therapy [4, 5] or metastatic breast cancer was discovered upon therapeutic splenectomy for ITP [6]. Here, we report a case of idiopathic thrombocytopenic purpura which presented after mastectomy for invasive ductal carcinoma.

## 2. Case Report

An 85-year-old Caucasian, postmenopausal female presented to the outpatient clinic with a palpable mass in her left breast. Initial diagnostic mammography identified spiculation and architectural distortion inferior to the 2.1 cm mass at the 12 o'clock position of the left breast. Targeted left breast ultrasound demonstrated two distinct hypoechoic lesions in the 12:00 axis of her left breast, which was classified as BIRADS-5. Ultrasound-guided biopsy revealed invasive ductal carcinoma (ER positive, PR positive, and Her2-Neu negative) in three different areas within her left breast. Left modified radical mastectomy with sentinel lymph node biopsy and axillary lymph node dissection was performed. Three of six sentinel lymph nodes were found to be positive on frozen section (one with macrometastasis and two with micrometastasis), and a full axillary lymph node dissection was performed.

The patient was convalescing satisfactorily until the afternoon of postoperative day one, when the output of her surgical drains became sanguineous and voluminous. A pressure dressing was applied to the chest wall. On postoperative day two, her hemoglobin had dropped to 8.8 g/dL from 13 g/dL preoperatively, and her platelet count decreased from 132,000/mm^3^ to 8,000/mm^3^. A fluid collection was noted along the length of her incision, primarily in the left axilla. She had extensive bruising along the incision and axilla, tracking downward on the abdomen. Despite these findings, the patient remained asymptomatic and hemodynamically stable. A peripheral blood smear demonstrated no cellular fragmentation. A review of the patient's current medications revealed no potential cause for a drug-induced thrombocytopenia, and a review of her past medical history revealed no other possible etiologies for thrombocytopenia. Given this, the patient was diagnosed with presumptive idiopathic thrombocytopenic purpura (ITP).

Beginning on postoperative day two, the patient began receiving 40 mg prednisone daily, and she received platelet transfusion. Following these measures, her platelet count increased to 87,000/mm^3^ and continued to improve thereafter. She was discharged from the hospital on postoperative day four in stable condition with a platelet count of 186,000/mm^3^. Her systemic steroids were then tapered over the course of four weeks.

## 3. Discussion

ITP is a relatively common autoimmune disease with an incidence of roughly 3.9 cases per 100,000 person-years [7]. While the etiology of primary ITP is currently unknown, causes of secondary ITP include other autoimmune disorders, infections, vaccinations, lymphoproliferative disorders, congenital immune deficiencies, and medications. Diagnosis of ITP requires a platelet count of less than 100,000/mm^3^ in the absence of splenomegaly with no evidence of an etiology for a secondary thrombocytopenia [8].

The incidence of ITP in patients with malignancies is unclear. The largest reported case series consists of ten patients with concurrent ITP and breast cancer [9]. Its mechanism in these patients is unknown but may be related to tumor infiltration of bone marrow, chemotherapy-induced or radiation-induced bone marrow hypoplasia, or platelet consumption due to disseminated intravascular coagulation [9]. Most of these patients with breast cancer who developed ITP demonstrated hormone receptor positivity with advanced progression of their cancers, specifically with bony metastases [10].

Standard first-line therapy for ITP is a 2- to 4-week course of corticosteroids dosed at 0.5–2 mg/kg, followed by a slow steroid taper [8]. Intravenous immunoglobulins or anti-RhD are sometimes required in conjunction with steroid therapy to stop acute bleeding. 80–90% of patients have a partial or complete response to steroid therapy [11]. Therapeutic options for refractory ITP include a splenectomy, as well as biologic agents such as rituximab, romiplostim, and eltrombopag [8], with 70–80% of patients achieving at least partial response following splenectomy [12].

## 4. Conclusion

We report the case of a patient whose ITP acutely presented twenty-four hours after a mastectomy and lymph node dissection for invasive ductal carcinoma of the breast. Her thrombocytopenia and acute bleeding responded well to standard corticosteroid therapy and conservative measures. Based on this case and other reported cases in breast cancer patients, ITP should be considered in the differential diagnosis for postoperative bleeding in patients undergoing mastectomy and lymph node dissection.
